# Supplementary material for: Mouse Model of STAT3 Mutation Resulting in Job’s Syndrome Diverges from Human Pathology
Source: Int J Mol Sci. 2025 Aug 8;26(16):7675. doi: 10.3390/ijms26167675 (PMC12387100; doi:10.3390/ijms26167675)
Supplement: Supplementary file 1 [file ijms-26-07675-s001.zip › ijms-3716391-supplementary.pdf]

International Journal of *Molecular Sciences*

MDPI

Supplementary Materials

Mouse Model of STAT3 Mutation Resulting in Job's Syndrome Diverges from Human Pathology

Jakub Jankowski [1,*], Jichun Chen [2], Gyuhyeok Cho [3], Sung-Gwon Lee [1], Chengyu Liu [4], Neal Young [2], Jungwook Kim [3] and Lothar Hennighausen [1]

[1] Section of Genetics and Physiology, Laboratory of Cellular and Molecular Biology, National Institute of Diabetes and Digestive and Kidney Diseases, US National Institutes of Health, Bethesda, MD 20892, USA; sunggwonl22@gmail.com (S.-G.L.); lotharh@niddk.nih.gov (L.H.)
[2] Hematology Branch, National Heart, Lung, and Blood Institute, National Institutes of Health, Bethesda, MD 20892, USA; chenji@nhlbi.nih.gov (J.C.); youngns@nhlbi.nih.gov (N.Y.)
[3] Department of Chemistry, Gwangju Institute of Science and Technology, Gwangju 61005, Republic of Korea; gyuhyeokcho@gm.gist.ac.kr (G.C.); jwkim@gist.ac.kr (J.K.)
[4] Transgenic Core, National Heart, Lung, and Blood Institute, US National Institutes of Health, Bethesda, MD 20892, USA; liuch@nhlbi.nih.gov
* Correspondence: jakub.jankowski@nih.gov

Supplementary Figure Descriptions

Figure S1: CRISPR/Cas9 genome modification resulted in an off-target deletion. (a) Visualization of the nucleotide (black) and amino acid (blue) sequence with marked intended amino acid change (red) and off-target deletion (dashes) resulting in generation of the $STAT3^{G656\_M660del}$ deletion. (b) Representative genotyping sequences of wildtype and heterozygous *G656_M660del* mouse. Different colors reflect nucleotides. Red line - $STAT3^{G656\_M660del}$ deletion.

Figure S2: $STAT3^{G656\_M660del}$ deletion causes significant changes in select serum parameters. (a) Serum IgE as measured by ELISA (b-j) CBC and flow cytometry results for select measures different between experimental groups. n = 7-8, *$p < 0.05$, **$p < 0.01$, bar = SEM

Figure S3: $STAT3^{G656\_M660del}$ deletion does not vastly alter overall immune landscape. (a-l) Remaining major immune components as measured by CBC and flow cytometry. n = 7-8, **$p < 0.01$, ***$p < 0.001$, bar = SEM

Figure S4: The immune phenotype persists after additional 2-month follow-up. (a-h) CBC and select measures depicted in Figure 1 (2 months) repeated in 4 months-old mice. n = 6 and 4 (WT and *G656_M660del*) *$p < 0.05$, **$p < 0.01$, bar = SEM

Figure S5: Representative gating strategy for flow cytometry – intracellular staining.

Figure S6: Representative gating strategy for flow cytometry – surface staining.

Table S1: Off-target CRISPR/Cas9 sites for the used sgRNA.

 https://doi.org/10.3390/ijms26167675

Figure S1

WT	ATC	ATC	ATG	GGC	TAT	AAG	ATC	ATG	GAT	GCG	ACC	AAC	ATC	CTG	GTG	TCT
	I	I	M	G	Y	K	I	M	D	A	T	N	I	L	V	S
Intended	ATC	ATC	ATG	GGC	TAT	AAG	ATC	ATG	CAT	GCG	ACC	AAC	ATC	CTG	GTG	TCT
	I	I	M	G	Y	K	I	M	H	A	T	N	I	L	V	S
G656_M660del	ATC	ATC	ATG	---	---	---	---	---	GAT	GCG	ACC	AAC	ATC	CTG	GTG	TCT
	I	I	M	G	Y	K	I	M	D	A	T	N	I	L	V	S

sgRNA:
CATCATGGGCTATAAGATCA

Donor Oligo:
CCAAGCAGCAGCTGAACAACATGTCATTTGCTGAAATCATCATGGGCTATAAGATCATG
CATGCGACCAACATCCTGGTGTCTCCACTTGTCTACCTCTACCCCGACATTCCCAAGGAGGAGGCATTTGG

(a)

WT

G656_M660del Forward

G656_M660del Reverse

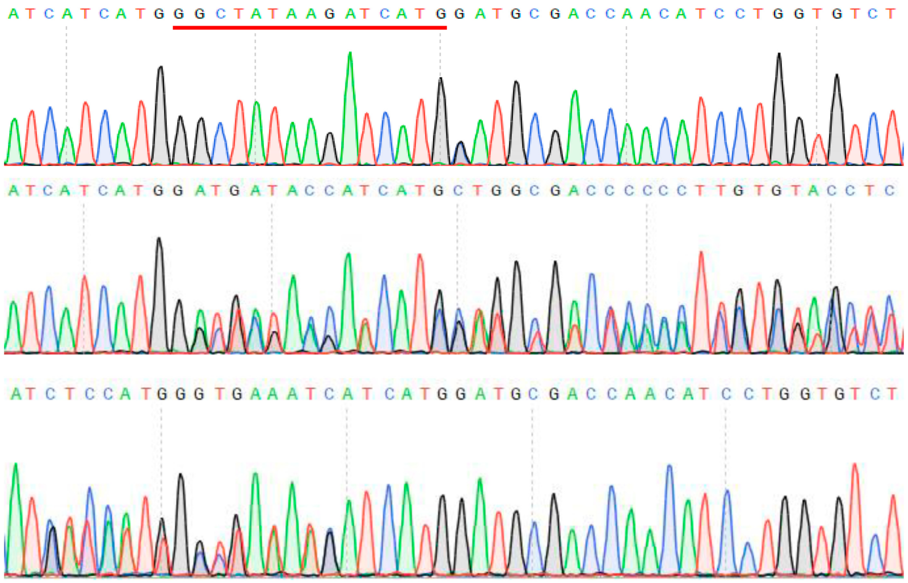

Forward primer:
AAGAAGGAATCACCAAGCCCTG

Reverse primer:
TCTGTACTCCCTTTCCTGCCTAC

(b)

Figure S2

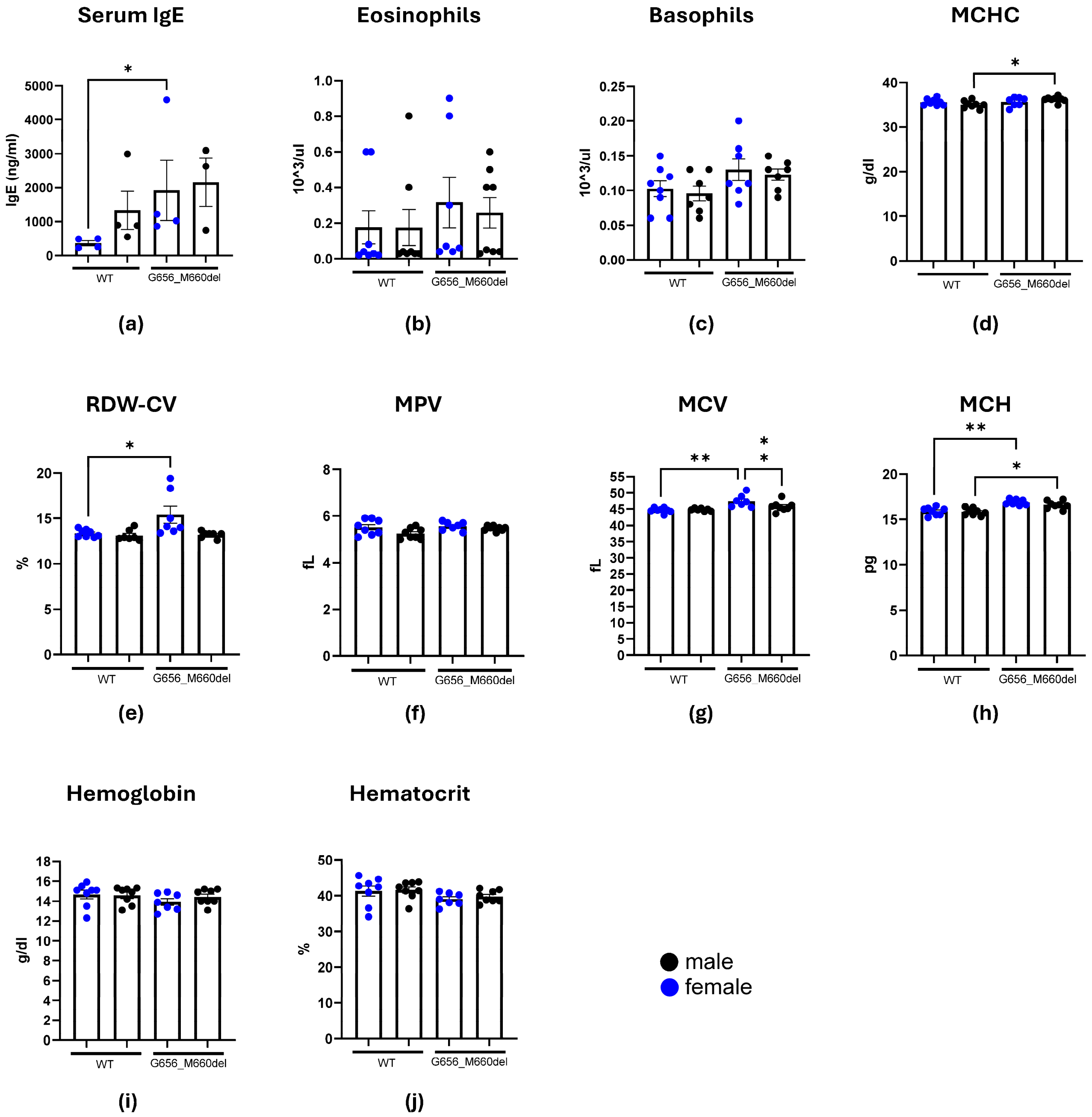
Serum IgE
IgE (ng/ml)
WT
G656_M660del
(a)
Eosinophils
10^3/ul
WT
G656_M660del
(b)
Basophils
10^3/ul
WT
G656_M660del
(c)
MCHC
g/dl
WT
G656_M660del
(d)
RDW-CV
%
WT
G656_M660del
(e)
MPV
fL
WT
G656_M660del
(f)
MCV
fL
WT
G656_M660del
(g)
MCH
pg
WT
G656_M660del
(h)
Hemoglobin
g/dl
WT
G656_M660del
(i)
Hematocrit
%
WT
G656_M660del
(j)
male
female

Figure S3

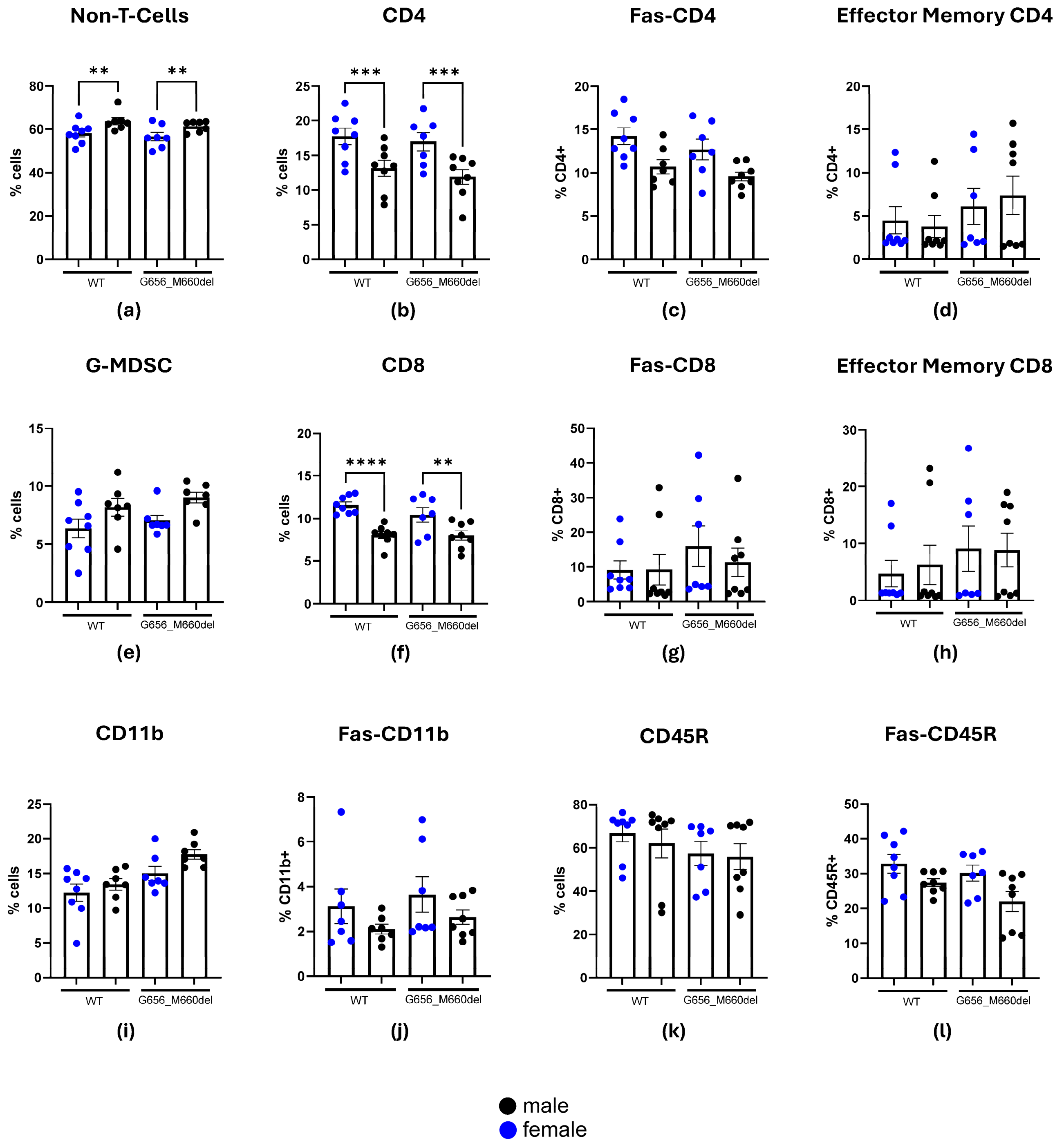
Non-T-Cells
CD4
Fas-CD4
Effector Memory CD4
G-MDSC
CD8
Fas-CD8
Effector Memory CD8
CD11b
Fas-CD11b
CD45R
Fas-CD45R
% cells
% CD4+
% CD8+
% CD11b+
% CD45R+
WT
G656_M660del
(a)
(b)
(c)
(d)
(e)
(f)
(g)
(h)
(i)
(j)
(k)
(l)
male
female

Figure S4

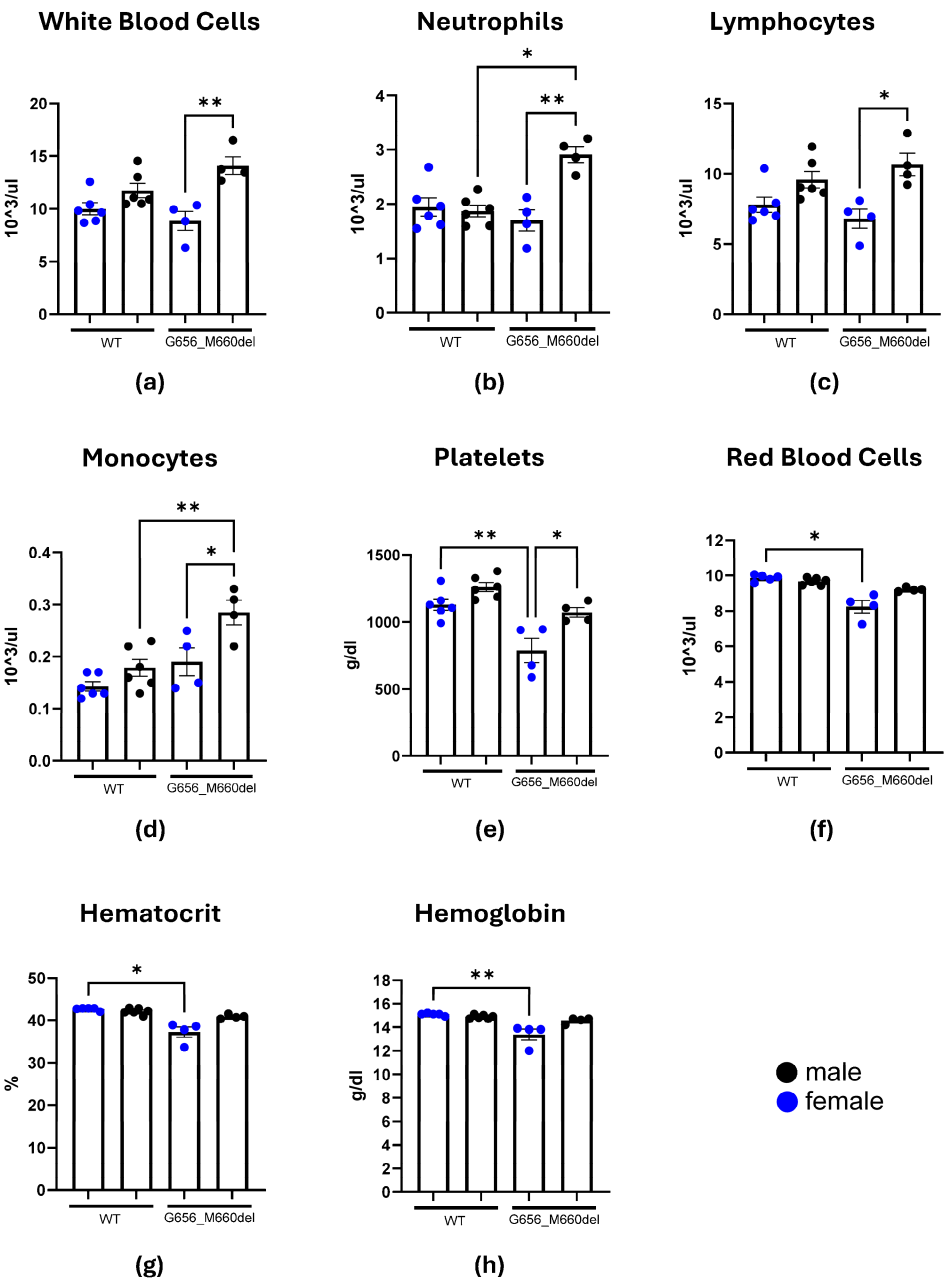
White Blood Cells
Neutrophils
Lymphocytes
Monocytes
Platelets
Red Blood Cells
Hematocrit
Hemoglobin
10^3/ul
g/dl
%
WT
G656_M660del
(a)
(b)
(c)
(d)
(e)
(f)
(g)
(h)
male
female

Figure S5

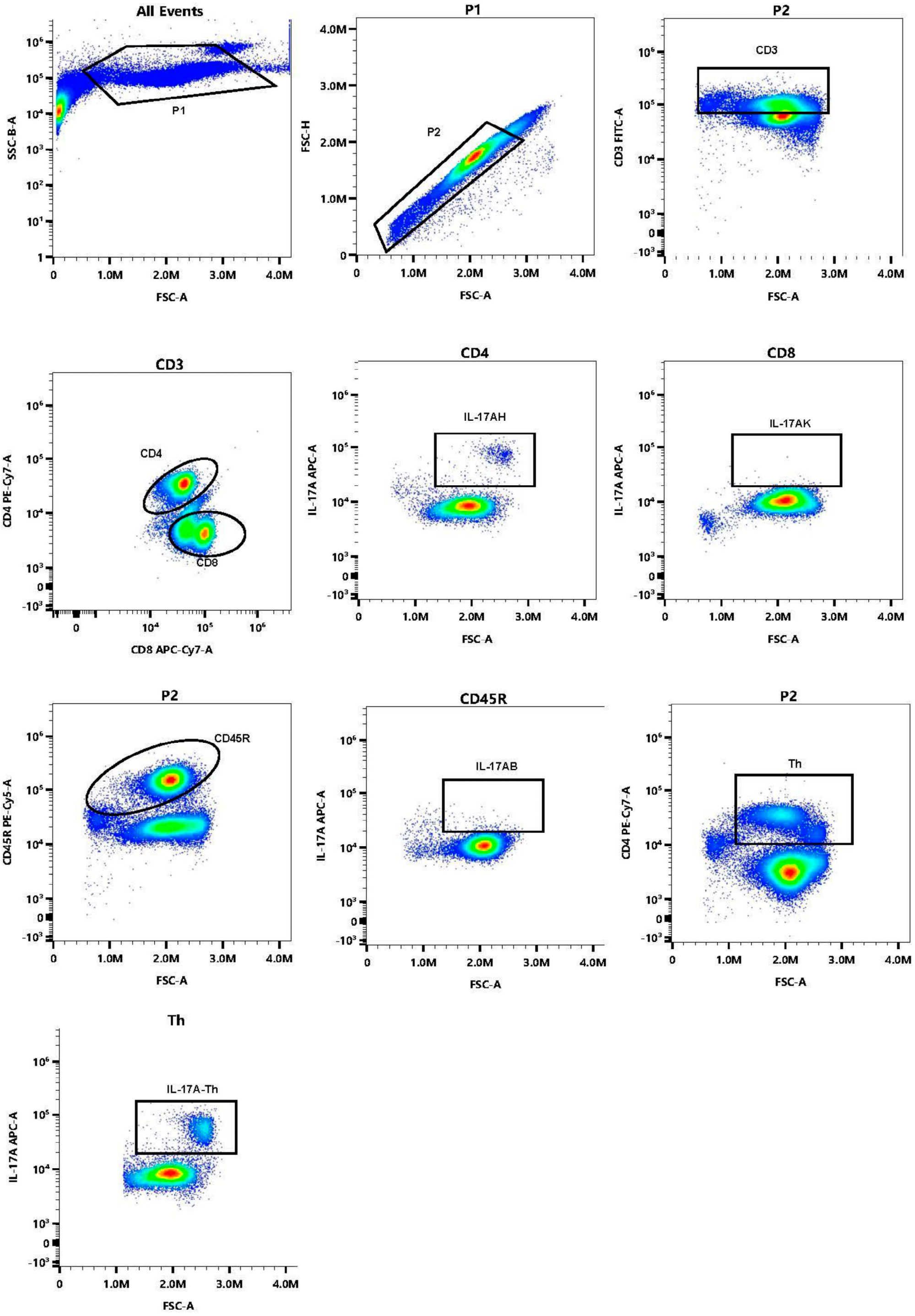
All Events
SSC-B-A
FSC-A
P1
FSC-H
P2
CD3 FITC-A
CD3
CD4 PE-Cy7-A
CD8 APC-Cy7-A
CD4
CD8
IL-17A APC-A
IL-17AH
IL-17AK
CD45R PE-Cy5-A
CD45R
IL-17AB
Th
IL-17A-Th

Figure S6

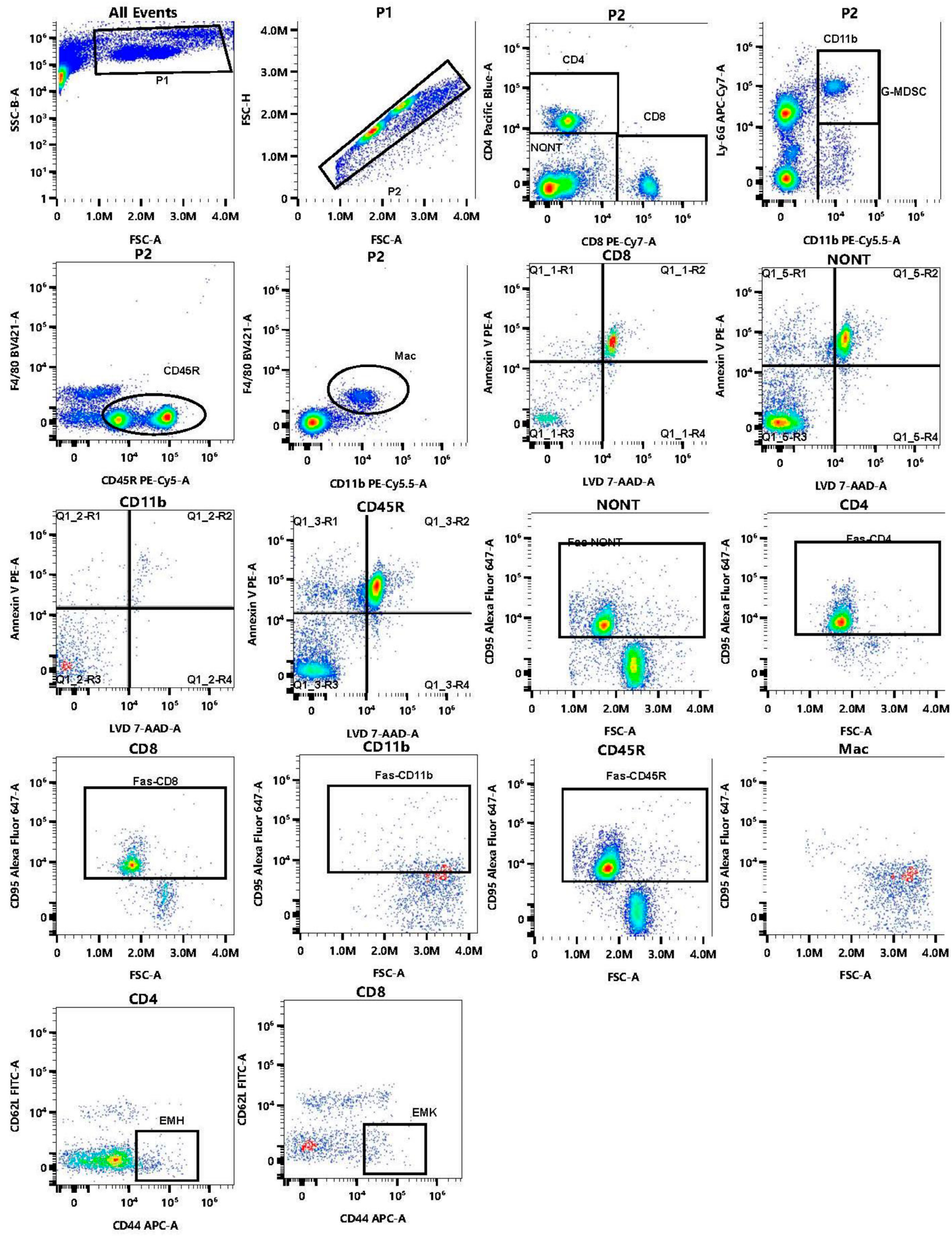
All Events
P1
SSC-B-A
FSC-A
P1
P2
FSC-H
FSC-A
P2
CD4
CD8
NONT
CD4 Pacific Blue-A
CD8 PE-Cy7-A
P2
CD11b
G-MDSC
Ly-6G APC-Cy7-A
CD11b PE-Cy5.5-A
P2
CD45R
F4/80 BV421-A
CD45R PE-Cy5-A
P2
Mac
F4/80 BV421-A
CD11b PE-Cy5.5-A
CD8
Q1_1-R1
Q1_1-R2
Q1_1-R3
Q1_1-R4
Annexin V PE-A
LVD 7-AAD-A
NONT
Q1_5-R1
Q1_5-R2
Q1_5-R3
Q1_5-R4
Annexin V PE-A
LVD 7-AAD-A
CD11b
Q1_2-R1
Q1_2-R2
Q1_2-R3
Q1_2-R4
Annexin V PE-A
LVD 7-AAD-A
CD45R
Q1_3-R1
Q1_3-R2
Q1_3-R3
Q1_3-R4
Annexin V PE-A
LVD 7-AAD-A
NONT
Fas-NONT
CD95 Alexa Fluor 647-A
FSC-A
CD4
Fas-CD4
CD95 Alexa Fluor 647-A
FSC-A
CD8
Fas-CD8
CD95 Alexa Fluor 647-A
FSC-A
CD11b
Fas-CD11b
CD95 Alexa Fluor 647-A
FSC-A
CD45R
Fas-CD45R
CD95 Alexa Fluor 647-A
FSC-A
Mac
CD95 Alexa Fluor 647-A
FSC-A
CD4
EMH
CD62L FITC-A
CD44 APC-A
CD8
EMK
CD62L FITC-A
CD44 APC-A

Table S1

Location	Number of mis-matches	Sequence (including mismatches)	Locus
NC_000068.8:70328280	3	CCTTGcTCaTATAaCCCATGATG	intron, *Gad1os* gene
NC_000069.7:34540781	3	CCCTGtTCcTtTAGCCCATGATG	~45 kb to nearest gene
NC_000069.7:114651895	3	CCATaATCTTATAGCtaATGATG	~45 kb to nearest gene
NC_000071.7:113507155	3	CAggATGGGCTAaAAGATCAGGG	~1.5 kb to nearest gene
NC_000075.7:62987885	2	CATCATGGGtTATtAGATCATGG	~12 kb to nearest gene
NC_000077.7:113460493	3	CCATGATgTaATAaCCCATGATG	intron, *Slc39a11* gene
NC_000078.7:57418757	3	tATCATGGGCTATAAGcTaATGG	intron, *Mipol1* gene
NC_000084.7:44527346	3	CtTCATGGGCTcTAAGAaCAAGG	intron, *Dcp2* gene
NC_000086.8:118459989	3	CATCATGGtCTgTAAGAaCAGGG	~375 kb to nearest gene
